# Identification of Ovarian Cancer Metastatic miRNAs

**DOI:** 10.1371/journal.pone.0058226

**Published:** 2013-03-12

**Authors:** Souriya Vang, Hsin-Ta Wu, Andrew Fischer, Daniel H. Miller, Shannon MacLaughlan, Elijah Douglass, Margaret Steinhoff, Colin Collins, Peter J. S. Smith, Laurent Brard, Alexander S. Brodsky

**Affiliations:** 1 Department of Molecular Biology, Cell Biology, and Biochemistry, Brown University, Providence, Rhode Island, United States of America; 2 Center for Computational Molecular Biology, Brown University, Providence, Rhode Island, United States of America; 3 Center for Genomics and Proteomics, Brown University, Providence, Rhode Island, United States of America; 4 Department of Computer Science, Brown University, Providence, Rhode Island, United States of America; 5 Program in Women's Oncology, Department of Obstetrics and Gynecology, Women and Infants' Hospital, Alpert Medical School of Brown University, Providence, Rhode Island, United States of America; 6 Department of Pathology, Women and Infants' Hospital, Alpert Medical School of Brown University, Providence, Rhode Island, United States of America; 7 Vancouver Prostate Centre, Vancouver, British Columbia, Canada; 8 Institute of Life Sciences, University of Southampton, Southampton, England; 9 Department of Obstetrics and Gynecology, Division of Gynecologic Oncology, Southern Illinois University School of Medicine, Springfield, Illinois, United States of America; H.Lee Moffitt Cancer Center & Research Institute, United States of America

## Abstract

Serous epithelial ovarian cancer (EOC) patients often succumb to aggressive metastatic disease, yet little is known about the behavior and genetics of ovarian cancer metastasis. Here, we aim to understand how omental metastases differ from primary tumors and how these differences may influence chemotherapy. We analyzed the miRNA expression profiles of primary EOC tumors and their respective omental metastases from 9 patients using miRNA Taqman qPCR arrays. We find 17 miRNAs with differential expression in omental lesions compared to primary tumors. miR-21, miR-150, and miR-146a have low expression in most primary tumors with significantly increased expression in omental lesions, with concomitant decreased expression of predicted mRNA targets based on mRNA expression. We find that miR-150 and miR-146a mediate spheroid size. Both miR-146a and miR-150 increase the number of residual surviving cells by 2–4 fold when challenged with lethal cisplatin concentrations. These observations suggest that at least two of the miRNAs, miR-146a and miR-150, up-regulated in omental lesions, stimulate survival and increase drug tolerance. Our observations suggest that cancer cells in omental tumors express key miRNAs differently than primary tumors, and that at least some of these microRNAs may be critical regulators of the emergence of drug resistant disease.

## Introduction

Serous Epithelial Ovarian Cancer (EOC) is an aggressive disease for which there are few effective biomarkers and therapies. EOC is often diagnosed after tumor cells have disseminated within the peritoneal cavity [1]. Despite the fact that metastases account for the majority of disease-related deaths, ovarian cancer metastasis remains poorly understood [1].

The purpose of this study was to identify features that may be important to establish metastases and to determine how these factors may affect chemotherapy responses. Advanced metastatic disease remains a daunting challenge to treat, most often leading to recurrent, drug resistant tumors. Metastases can be enriched for a distinct mutational spectrum compared to primary tumors [2], [3], [4]. Comparing primary and metastatic tumors has generated important insights into disease progression in both animal models [5] and in patients [2]. To improve treatment of metastatic disease, it is vital to understand the genes and pathways emerging in metastases that may not be present in primary tumors. Although metastatic potential can be predicted based on the primary tumor [6], [7], this observation is not mutually exclusive with the possibility that key features emerge in metastases that are not observed in primary tumors. For example, the new microenvironment can induce significant phenotypic changes to cancer cells, including changes to metabolic activity in the omentum [8], and increased drug resistance [9].

Previous mRNA expression studies examining matched ovarian primary and metastatic tumors from the same patient, support a ‘primary tumor predisposition’ model [6], [10], [11], [12]. mRNA expression data using early generation microarrays suggest there are few significant expression differences between omental lesions and primary tumors [13], [14], [15], however, numerous studies have described differential expression of key regulatory factors between primary tumors and metastases, including E-cadherin [16], MMPs [17], [18] and integrins [19]. To address this apparent discrepancy and to gain new insights into the state of cancer cells in metastases, we profiled miRNA expression in matched pairs of primary serous epithelial ovarian (EOC) tumors and omental lesions. miRNA expression profiling identifies miR-150 and miR-146a to be up-regulated in omental metastases. We find that miR-150 and miR-146a promote spheroid formation and increase the fraction of residual surviving cells after cisplatin exposure. These observations suggest that higher expression of miR-146a and miR-150 in omental lesions may lead to more aggressive, chemoresistant disease.

## Results

We identified 9 Stage IIIC serous epithelial ovarian cancer patients with pairs of primary and omental metastatic tumor specimens (Figure S1, Table S1). All patients were post-menopausal (>55 years old at time of diagnosis) and had metastatic disease in the omentum. We measured miRNA expression using Taqman qPCR array cards in the 9 pairs of tumors. Each tumor had >70% cancer cells, and good RNA quality (Agilent Bioanalyzer RIN>5). Our focus is to understand the changes manifesting during disease progression, and therefore we have focused on comparing the metastases to the primary tumors and did not consider normal ovarian epithelial cells.

### Identification of miRNAs differentially expressed between primary and metastatic tumors

**Figure 1:**
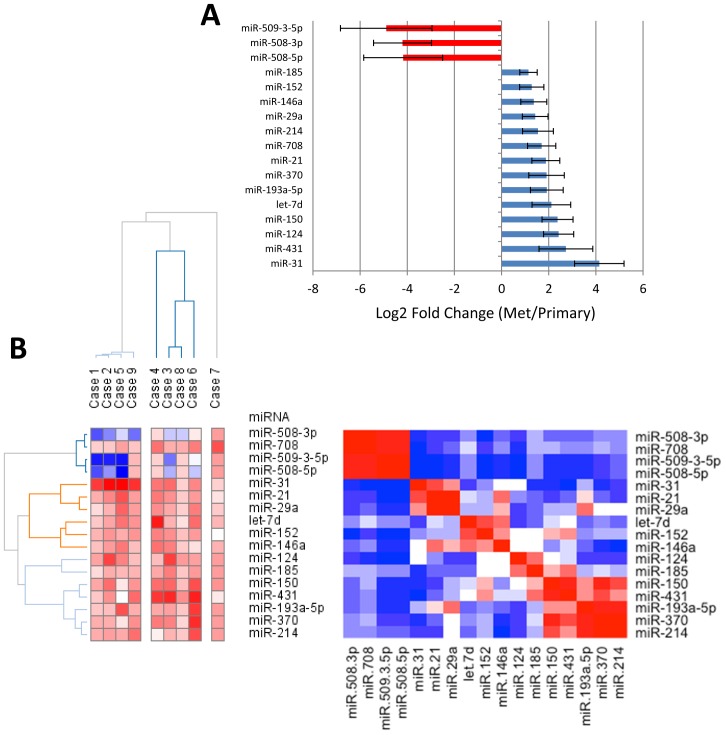
miRNA expression profile of primary and metastatic ovarian tumors. **A**. miRNA expression profiling by Taqman qPCR arrays identifies 17 miRNAs differentially expressed between 9 pairs of primary tumors and omental lesions. miRNAs with p<0.05 (paired t-test) were selected. The expression level is presented as the mean +/− standard error of the mean (s.e.m.) of the fold change using the ΔC_t_ method relative to U6 snRNA. Red, lower expression in metastases, blue, higher expression in metastases. **B**. Left, unsupervised hierarchical clustering of the fold changes of the analyzed patients. Right, association plot analysis of miRNA expression identifies miRNA clusters indicated in red. Clustering was performed in Gene-E ]50].

We measured 377 miRNAs using ABI Taqman qPCR arrays, specific for mature miRNAs [20], in 9 matched primary and metastatic human tumors. 180 miRNAs are expressed, in at least two tumors, with no global up- or down-regulation of these miRNAs between the primary and metastatic tumors. Figure 1A summarizes the miRNAs with large recurring expression differences as identified by a paired t-test (Figure S2). We tested the expression of miR-146a and miR-150 in assays targeting just these miRNAs in two pairs of patients to confirm that the Taqman assays are specific for these miRNAs with no cross-talk from the other 376 assays (Figure S3).

Hierarchical clustering of the fold change between primary and metastatic tumors for the 17 miRNAs shows three distinct groups (Figure 1B). Patients were clustered with similar fold changes in miRNA expression (Figure 1B, left panel). Distinct miRNA groups are more easily visualized when the miRNAs are clustered independent of the patients (Figure 1B, right panel). Because miR-146a, miR-21 and miR-150 are representative of the three major up-regulated clusters, we decided to focus our efforts to understand their possible functions in ovarian cancer. We found that the expression of miR-21, miR-146a, miR-150 is negatively correlated with their predicted mRNA targets (Figure S4), suggesting that these miRNAs are actively suppressing mRNA expression in metastases compared to primary tumors. We chose to focus on miR-146a and miR-150 because these two miRNAs have not previously been examined in ovarian cancer to our knowledge.

**Figure 2:**
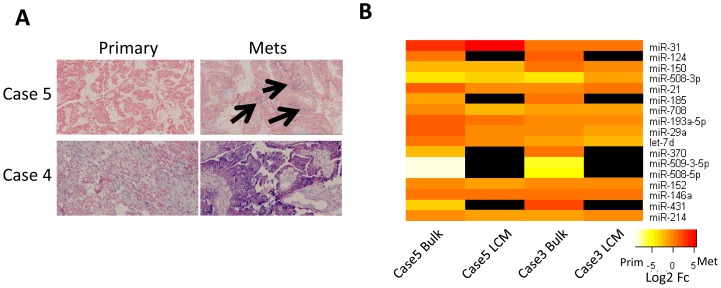
Validation of miRNA expression. **A**. *In situ* hybridization of miR-21. Cancer cells are stained red by Nuclear Red. Higher expression in omental metastases is observed in each case even with both relatively high and low miR-21 expression in the primary tumor. The arrows indicate regions of miR-21 expression co-localizing with Nuclear Red staining. **B**. Laser Capture Microdissection (LCM) of two cases reveals miRNAs likely expressed in cancer cells. The heat map shows the fold change for the 17 miRNAs identified in the bulk tumor screen. Similar patterns of differential expression are observed for the miRNAs expressed in cancer cells as observed in bulk tumor. Black indicates that the miRNA was not detectable in the LCM isolated cancer cells.

To determine if the expression changes originate from cancer cells or stroma, using an orthogonal assay, we performed *in situ* hybridization (ISH). miR-21 is expressed in both cancer and stroma cells, and up-regulated in omental lesions, consistent with the Taqman qPCR screen (Figure 2A). By ISH, we observe that the absolute expression levels are variable, but all 8 omental lesions tested show increased miR-21 cancer cell expression compared to the corresponding primary tumor (Figure 2 and Figure S5). In our hands, ISH sensitivity is poor and depends on the quality of the probe, and we were unable to obtain reliable signal for other miRNAs, even after exhaustive examination of key variables including hybridization temperature, proteinase K concentration, and probe concentration. These observations suggest that miR-21 originates from both cancer and stroma cells, and that miR-21 expression increases in omental metastases in cancer cells.

In order to pursue a more global analysis, we enriched for cancer cells by Laser Capture Microdissection (LCM) of H&E stained cancer cells, and performed Taqman qPCR arrays from two cases. 11 of the 17 miRNAs, identified in the bulk tumor screen (Figure 1A), are expressed in LCM enriched cancer cells, and the differential expression between primary tumors and omental lesions is qualitatively the same (Figure 2B, Figure S6). These observations suggest that the observed change in expression likely originates in cancer cells for these 11 miRNAs (Figure 2B). miRNAs not expressed in cancer cells, but with large expression changes in the bulk tumor, such as miR-124 and miR-370, may indicate the presence of specific types of stroma cells such as fibroblasts or immune cells. We were initially intrigued by miR-509-3-5p, miR-508-3p, and miR-508-5p as these were the only miRNAs down-regulated in metastases in the bulk tumor measurements. However, these three miRNAs are not expressed in the LCM enriched cancer cell populations (Figure 2B), and are not significantly expressed in the tested ovarian cancer cell lines (Figure S7), and thus were not considered further.

Although LCM selected cancer cells are not 100% cancer cells, these observations strongly suggest that miR-146a and miR-150 are likely expressed in cancer cells and that their expression is up-regulated in omental metastases. Importantly, we find expression of these miRNAs in H&E stained, LCM enriched cancer cells in both primary and metastatic tumors, consistent with their likely expression in cancer cells. TCGA has found that miR-150 and miR-146a are expressed at low levels in most primary tumors [21], consistent with our observations.

### miR-150 and miR-146a promote spheroid formation

Taqman qPCR arrays revealed that 8 of the 17 metastatic miRNAs (Figure 1A) are expressed in proliferating OVCAR-8 and SKOV-3 cells (Figure S7) and in cancer cells in the human tumors (Figure 2B). miR-146a is expressed at relatively low levels and miR-150 is not significantly expressed as it was only detected above the recommended C_t_ thresholds in the proliferating ovarian cancer cell lines tested.

**Figure 3:**
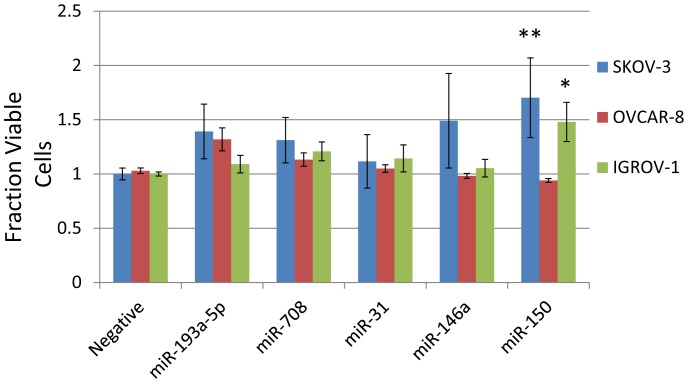
miR-150 promotes growth of ovarian cancer cells in monolayer. 24 hours after transfection with 50 nM pre-miRs, cells were plated into 96 well plates and grown for 4 days. Viable cells were determined by Wst-1 normalized to cells transfected with negative control pre-miRs. Error bars (s.e.m.) represent indendent biological triplicates. Each replicate consistes of consisting of three wells in a 96 well plate. **, p<0.01, *, p<0.05 by Student's t-test.

We hypothesized that miRNAs up-regulated in the omental lesions would stimulate growth as part of their ability to promote aggressive disease. We tested miRNAs expressed in cancer cells in the tumors (Figure 2B) that are also modestly expressed in ovarian cancer cell lines to avoid over-expressing miRNAs at supra-physiological concentrations including miR-150, miR-146a, miR-708, and miR-193a-5p. To model the higher miRNA expression observed in omental lesions, we ectopically expressed synthetic pre-miRs and performed gain of function screens in cell viability and cisplatin sensitivity assays. Transfection of pre-miRs lead to high overexpression of miR-146a (Figure S8, while miR-150 is modestly expressed compared to U6 snRNA (ΔC_t_ ∼3). Ectopic expression of miR-150 modestly increased the number of viable cells in SKOV-3 and IGROV-1 over four days, but not in OVCAR-8 cells (Figure 3). None of the other pre-miRs induced significant, reproducible effects on growth in more than one cell line.

**Figure 4:**
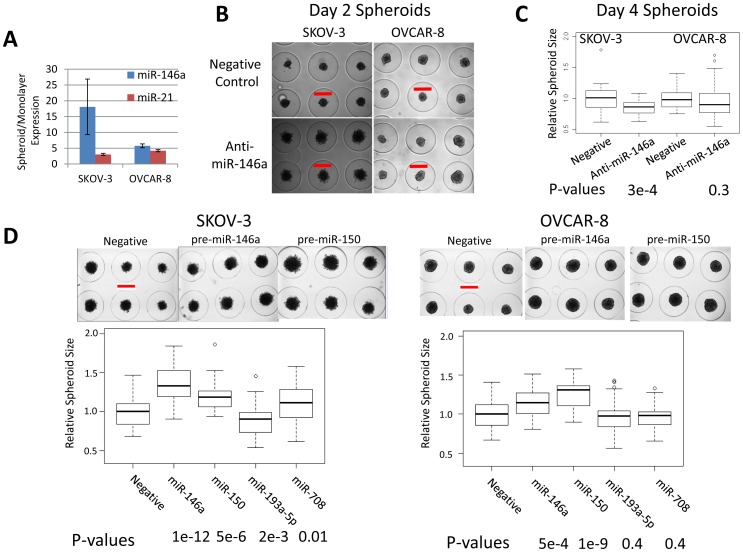
miR-146a and miR-150 enhance spheroid formation. 24 hours after transfection with anti-miR LNA inhibitors or pre-miR mimics as indicated, 700/µl SKOV-3 or 600/µl OVCAR-8 cells were seeded in 35 well agarose micromolds with one spheroid forming in each well. **A**. miR-21 and miR-146a are up-regulated in 4 day SKOV-3 and OVCAR-8 spheroids detected using Taqman qPCR array cards in two replicates. Error bars are s.e.m. **B**. Inhibition of miR-146a with 10 nM LNA delays spheroid formation and leads to more amorphous and looser formed spheroids in SKOV-3 and OVCAR-8 after 2 days. Red bar is 400 µm. **C**. Box and whisker plot shows that inhibition of miR-146a with LNAs significantly reduces spheroid size after 4 days in SKOV-3, and modestly in OVCAR-8. Representative expression shown from four replicates. **D**. Ectopic expression of miR-150 and miR-146a significantly enhances spheroid formation after 4 days. SKOV-3 and OVCAR-8 cells were transfected with 50 nM of pre-miR miR-150 and miR-146a pre-miRs before spheroid formation. SKOV-3 or OVCAR-8 cells were transfected as indicated. Representative spheroids are shown. Red bar is 400 µm. Box and whisker plots of the size distribution of 45 spheroids from a representative experiment. Experiment was reproduced three times. P-values determined by Student's t-test.

Spheroids model multicellular aggregates in the ascites of ovarian cancer patients that establish metastases [22]. Spheroids are a more accurate representation of tumors and increasing evidence suggests that drug responses are better modeled in spheroids than in monolayer culture [23], [24], [25], [26]. Spheroid formation typically requires a minimum number of cells, followed by spontaneous aggregation, survival under anchorage independent conditions, and compaction to strengthen the survival of the aggregate [27]. To evaluate miRNA function in spheroids, we form uniform spheroids by seeding cells into agarose micromolds [28]. Interestingly, all the expressed miRNAs are up-regulated in 3D spheroids compared to monolayer using a Taqman array card (Figure S4). We tested miR-146a and miR-21 expression by Taqman qPCR, using primers targeting only these two miRNAs, and reproducibly observe up-regulation of these two miRNAs in spheroids (Figure 4A). To test if up-regulation of miR-146a is important for spheroid formation, we inhibited miR-146a with a Locked Nucleic Acid (LNA) inhibitor. miR-146a inhibition causes amorphous, more loosely formed spheroids in both SKOV-3 and OVCAR-8 cells after two days (Figure 4B), compared to the more compact spheroids formed in the negative controls. The effect is more dramatic in SKOV-3 than Ovcar-8. Because of the amorphous nature of these early forming day 2 spheroids, we could not reliably determine their sizes. This observation suggests that early spheroid formation is impacted by a reduction of miR-146a activity. After 4 days, when control spheroids have more fully formed after undergoing compaction, inhibition of miR-146a with a LNA inhibitor reduces spheroid size, compared to a negative control LNA, in SKOV-3, but only modestly in OVCAR-8 (Figure 4C). 45 spheroids were measured in each replicate, and three independent replicates were performed. The size of each spheroid was determined by ImageJ and the size distribution of spheroids is represented with box plots (Figure 4C and 4D). Spheroid formation can be challenging due to slight differences in the number of cells used to seed each spheroid. With agarose molds, we evaluate a large number of spheroids to overcome concerns with errors in cell number used to seed the spheroids in each well allowing for distinct miRNA dependent differences to be observed.

Consistent with increased expression of miRNAs in spheroids being critical for spheroid formation, higher miR-150 and miR-146a expression promote larger spheroids in SKOV-3 and OVCAR-8 (Figure 4D). Introduction of miR-150 and miR-146a show the most consistent and largest effects on spheroids compared to the negative control and the other pre-miRs tested. For miR-146a, we observe smaller spheroids when miR-146a is inhibited (Figure 4C) and larger spheroids upon ectopic expression (Figure 4D) after 4 days of spheroid formation. Together, these observations, support miR-146a promoting spheroid formation.

### miR-150 and miR-146a increase cisplatin tolerance

**Figure 5:**
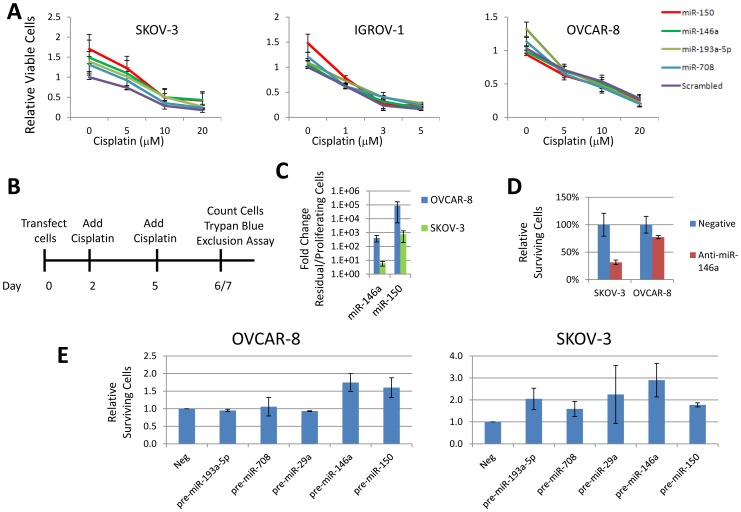
Metastatic miRNAs increase surviving cells. **A**. Treatment of cells with pre-miR-150 mimic modestly increases the cisplatin IC50 in SKOV-3 and IGROV-1, but not OVCAR-8 cells. Wst-1 assays were performed 48 hours after cisplatin treatment. Graph shows average of 3 biological replicates. Error bars represent s.e.m. **B**. Schematic of cisplatin survival assay. Cells are treated twice with high concentrations of cisplatin leading to survival by approximately 1% of the cells. **C**. miR-150 and miR-146a are up-regulated in surviving cells after 6 days of 50 µM cisplatin in SKOV-3 and 7 days of 30 µM cisplatin in OVCAR-8 cells compared to untreated, proliferating cells. Data are in duplicate and error bars are s.e.m. The fold change for miR-150 is very large because miR-150 was not detectable in proliferating cells. C_t_ was set to maximum cycle tested, 40, to estimate the fold change. **D**. Inhibition of miR-146a with 10 nM LNA inhibitor significantly reduces the number of residual cells in SKOV-3 and modestly reduces the surviving cells in OVCAR-8 after 6 days of 50 µM cisplatin in SKOV-3 and 7 days of 30 µM cisplatin in OVCAR-8 cells. The surviving viable cells were determined by trypan blue exclusion assay. Biological triplicate experiments are shown. Error bars are s.e.m. **E**. Transfection of 50 nM pre-miR-146a and pre-miR-150 increase long term survival after 6 days of 50 µM cisplatin in SKOV-3 and 7 days of 30 µM cisplatin in OVCAR-8 cells.

Once metastases are established, ovarian cancer is very difficult to treat as recurrent resistant tumors often re-emerge after initial chemotherapy. Changes in miRNA expression may indicate a different physiological state for the cancer cells in the metastases affecting how these lesions would respond to chemotherapy. We tested the effect of increased expression of four miRNAs, up-regulated in omental lesions, on cisplatin sensitivity. Dose dependent studies using Wst-1 assays reveal that higher expression of miR-150 modestly increases the cisplatin IC_50_ in SKOV-3, but not OVCAR-8 and IGROV-1 (Figure 5A). Other miRNAs, such as miR-146a, affected either SKOV-3 or IGROV-1, but not both in a statistically significant manner.

Careful examination of the cells in monolayer culture during cisplatin treatment suggested that healthier cells survived with higher expression miR-146a and miR-150 in high cisplatin concentrations. The dynamic range of remaining cells is below the detection limit of the Wst-1 assay. Recent studies have identified reversible drug tolerant quiescent cells that survive lethal concentrations of drugs [29]. Thus, examination of surviving cells represents an alternative model to examine how cancer cells survive chemotherapy. To test if the metastatic miRNAs promote survival in lethal doses of cisplatin, we examined miRNA expression in surviving residual cells in monolayer culture exposed to lethal doses of cisplatin for 6–7 days. We find that miR-150 and miR-146a are significantly up-regulated in the surviving cells compared to the proliferating population (Figure 5C). miR-150 is undetectable in OVCAR-8 cells and below the ABI recommended threshold in SKOV-3 cells (C_t_∼36 cycles) in proliferating cells, but becomes significantly expressed in residual surviving cells in both cell lines, suggesting a possible role in surviving cisplatin. To test if miR-146a affects the ability of ovarian cancer cells to survive long-term cisplatin treatment, inhibition of miR-146a with LNAs reduces the number of viable surviving residual cells modestly in OVCAR-8, and more significantly in SKOV-3 cells (Figure 5D). Consistent with their increased expression in the surviving cells, we find that higher expression of miR-150 or miR-146a significantly increases the number of surviving viable cells by trypan blue exclusion assay, six (SKOV-3) or seven (OVCAR-8) days after addition of lethal concentrations of cisplatin (Figure 5E). Trypan blue is an indicator of the number of viable cells and reflects the increased number of surviving cells we observed visually. Not all the ectopically expressed miRNAs improve survival suggesting that the effects of miR-150 and miR-146a are specific. These observations suggest that increased expression of miR-150 and miR-146a promote survival, or at least, delay cisplatin induced cell death, in ovarian cancer cells.

## Discussion

Our key findings demonstrate that metastatic tumors up-regulate specific miRNAs compared to their primary tumors and that, among these miRNAs, miR-146a and miR-150 promote 3D spheroid formation and increase tolerance to cisplatin in ovarian cancer cells, suggesting a role for these miRNAs for survival in specific conditions. We observe significant common recurrence of differential regulation of 17 miRNAs, suggesting that the requirements to adapt to the omentum are very similar in the majority of EOC patients. Together, these data support the idea that omental lesions are enriched for features of aggressive disease, which also mediate the patient's response to chemotherapy. Some of these features, such as miR-146a and miR-150 expression may be unique to metastases, as they are very lowly expressed in most primary tumors in our dataset and in TCGA [21]. Low expression of these miRNAs in primary tumors is associated with poor overall patient survival [21]. Because these miRNAs are often up-regulated in omental lesions and highly correlated with expression in primary tumors (Pearson = 0.7 for miR-150 and 0.78 for miR-146a), we predict that higher expression in metastases will be associated with shorter overall survival and disease progression.

One of the goals of this study was to understand how similar or different primary ovarian tumors are from omental metastases. Previous efforts comparing these tumors have applied microarray technologies to examine mRNA expression levels. Using strict thresholds, relatively small differences between primary and metastatic tumors were reported [15]. However, a number of studies report significant differences by other methods including immunohistochemistry of E-cadherin [19], MMPs [17] and the recent finding of adipocyte signaling affecting cancer cells in the omentum [8]. miRNAs have emerged as key regulators of cell fate and numerous miRNA profiling studies suggest that miRNAs may have a larger dynamic range in their expression differences across tissues allowing for the identification of distinct tissue and tumor specific expression signatures.

Because the stroma cells differ between the two tumors, some of the large miRNA expression differences could originate from these cells. To identify which, if any, miRNA expression differences originate from cancer cells, we performed two experiments. We were able to detect miR-21 by ISH, which shows increased miR-21 expression in H&E stained cancer cells (Figure 2A and Figure S5). These data also show that some increased miR-21 expression in some patients originate from stroma cells. To perform a more comprehensive analysis, we combined Taqman qPCR arrays with LCM to examine an enriched cancer cell population. These data reveal that some of the miRNAs identified in the bulk screen (miR-370, miR-124, miR-508, miR-509) are likely not expressed in cancer cells as they were not readily detected in the LCM enriched population. On the other hand, 11/17 of the miRNAs, including miR-146a and miR-150, identified in bulk tumor are expressed and maintain similar expression differences in the LCM enriched cancer cells.

Expression profiling from both bulk tumor and LCM enriched cancer cell populations as well as ISH data suggest significant up-regulation of miR-21 in metastases compared to primary tumors. miR-21 is well-known to be an anti-apoptotic, pro-survival miRNA in many cancers, including ovarian [30], and our preliminary observations also support miR-21′s role in promoting spheroid formation (data not shown). These observations support the concept that omental metastases may be selected to be more resistant to chemotherapy having survived escape from the primary tumor.

miRNAs often down-regulate expression by binding the 3′UTR of mRNAs. The effect on mRNA stability and total RNA levels is often modest [31], [32], assessment of mRNA expression changes can be difficult to observe. To gain insight into how up-regulated metastatic miRNAs are mediating proliferation and cisplatin response, we evaluated their predicted targets. We find that miR-21, miR-146a and miR-150 mRNA targets are significantly down-regulated compared to randomly selected equivalently sized gene sets (Figure S4), consistent with these miRNAs actively repressing mRNAs in metastatic tumors.

miR-150 is most well-known for its role in regulating B-cell differentiation and the timing of expression is critical for its proper role in promoting B cell development [33], [34]. Recent reports suggest that miR-150 can either promote or inhibit tumors [35], [36], [37], [38], highlighting the common theme of context dependent functions of miRNAs [39], [40]. We do not observe inverse correlation with the expression of previously identified miR-150 targets P2RX7 [41] or EGR2 [35] in our primary/metastatic tumor data.

miR-146a has been identified as a tumor suppressor through down-regulation of the NFkB activators IRAK1 and TRAF6 [42], [43]. However, we find that IRAK1 and TRAF6 are not expressed in SKOV-3 or OVCAR-8 by qPCR (data not shown). In some contexts, miR-146a is oncogenic, by suppressing BRCA1 [44] or FAS [45]. We did not observe significant reduction of BRCA1, BRCA2, or FAS expression upon miR-146a ectopic expression (data not shown). Suppression of BRCA1 would not make sense with increased survival, as decreased BRCA1/BRCA2 mediated DNA repair functions are associated with higher cisplatin sensitivity [46]. Thus, miR-146a appears to work through a novel mechanism in ovarian cancer cells to increase survival.

We hypothesized that changes in miRNA expression in metastases compared to primary tumors may indicate functions in the metastatic environment that differ from the primary tumor environment. To begin to model how these miRNAs may support sustained growth and survival of metastatic tumors, we embarked on a series of functional experiments using established ovarian cancer cell lines. We used gain and loss of function studies in cisplatin cell viability assays to find no significant effects of miR-146a and miR-150 on drug sensitivity or growth in ovarian cancer cells. Preliminary studies testing migration did not reveal significant miRNA dependent effects (data not shown). However, we find that miR-146a and miR-150 mediate the formation and size of spheroids. As cancer cells escape the primary tumor and enter the peritoneal cavity, they often form aggregates from 50–750 µm in size. Spheroids resemble these aggregates isolated from patients [47] and resemble xenograft tumors better than monolayer culture [48]. Some of the changes such as increased expression of integrins seen in established metastases are also observed in these spheroids (data not shown) and may reflect the community effect more reminiscent of human disease [19], [47], [49]. Our observations that miR-146a is up-regulated in human omental metastases, with a concomitant decrease in predicted mRNA targets, and spheroids in conjunction with gain and loss of function assays all suggest an important role for miR-146a in formation and maintenance of metastases. These data also support a role for miR-150, though without loss of function data, the conclusions based on the functional experiments are not as strong. Together with the cisplatin tolerance assay, these data support the possibility that miR-146a and miR-150 are need to support survival under stressed conditions such as spheroid growth, high concentrations of cisplatin treatment, and adaptation to new environment conditions during dissemination in patients.

A caveat of this study is that these cell lines may not recapitulate key features of cancer cells in tumors, including expression of miR-150 seen in ovarian tumors. Our inability to properly model ovarian cancer *in vitro* or *in vivo* may be obscuring additional functions of these miRNAs up-regulated in omental metastases. Short term cultures of newly derived cell lines or examination of miRNA function in animal models may be necessary to identify additional functions of these miRNAs in metastasis. These data highlight how some miRNAs may be important for survival in specific conditions and are thus selected for increased expression in metastases. Future studies examining miR-146a and miR-150 using *in vivo* models and co-culture systems may help provide insight into the functions of these miRNAs.

One of the major challenges in treating advanced metastatic disease is the relatively rapid appearance of recurrent, chemoresistant tumors. Our data support the hypothesis that cancer cells in omental lesions develop into a state distinct from the primary tumor as defined by differential expression of specific miRNAs. This hypothesis suggests that deeper examination of metastases is necessary to improve treatment of ovarian cancer, as distinct pathways may be activated or repressed, leading to different effects on growth and survival that impact chemotherapy response. The importance of many of these factors may not be readily detectable in primary tumors. Our examination of miR-146a and miR-150 function supports this hypothesis by suggesting that these miRNAs have significantly increased expression in metastases, in 3D spheroids, and in surviving cancer cells. These observations support future examination of larger patient cohorts to test if specific changes in omental metastases indicate patient survival better than expression changes in primary tumors. Pharmacological inhibition reducing miR-146a and miR-150 levels may be a novel approach to reduce the likelihood of the emergence of recurrent drug resistant tumors.

## Materials and Methods

### Patient material

Serous epithelial ovarian tumors were collected from de-identified cases using protocol #08-0095 approved by the Institutional Review Board of the Women's and Infants Hospital of Rhode Island. All patients were over age 55 at the time of diagnosis, stage III or later, with evidence of metastatic disease from imaging, and all tumors were chemo-naïve. A pathologist specializing in gynecological cancers examined all specimens (MS). Samples were snap frozen in liquid nitrogen with no fixation.

### Immunohistochemistry

Immunhistochemistry was performed on 4 µm slices of formalin fixed paraffin embedded (FFPE) tissue with the following antibodies: CA-125 (Dako) and monoclonal Cytokeratin (Dako). IHC was performed using a Dako EnVision™ FLEX detection system according to manufacturer's instructions.

### RNA Isolation and MicroRNA expression analysis by Taqman low-density array

Tumor tissue with >70% cancer cells was homogenized with a Tekmar Tissumizer (Cincinnati, OH). RNA was purified using miRNeasy kit (Qiagen, Valencia, CA) following the manufacturer's instructions. 500 ng of RNA was reverse-transcribed using the Taqman MicroRNA Reverse Transcription Kit and the Megaplex RT primer Human Pool A (Applied Biosystems). The cDNA was diluted and loaded on to a Taqman Human miRNA Array card A v. 1.0 (Life Technologies), which contains probes for 377 distinct miRNAs. The Array cards were run on an ABI HT7900 qPCR instrument. C_t_ values were obtained for all miRNAs represented on the cards and fold changes in expression were calculated using the ΔC_t_ method relative to U6 snRNA. For assays targeting individual miRNAs, 250 ng of total RNA from the bulk tumor or cell lines was reverse transcribed with only the primers for the miRNA or U6 snRNA. Equal amounts of cDNA were used for Taqman assays and analyzed using the ΔC_t_ method relative to U6 snRNA.

Hierarchical clustering was performed using GENE-E [50] with distances determined by Pearson Correlation and average linkage.

### Laser Capture Microdissection and miRNA measurement

Frozen tumor samples were placed into tissue cryomolds (25 mm×20 mm×5 mm, Sakura Finetek USA, INC., Torrance, CA, USA) and submerged in optimal cutting temperature (OCT) compound (Sakura). The samples were allowed to solidify on dry ice and then placed inside a 50 mL conical (Corning Inc., Corning, NY, USA) and stored in a −80°C freezer. Microdissection was performed using the Arcturus PixCell IIe LCM system (Applied Biosystems, Bedford, MA, USA) as detailed by the manufacturers protocol and 2000–3000 cancer cells were collected. Total RNA was extracted using RNeasy minElute (Qiagen) as per manufacturer's protocol with modifications to capture miRNAs during RNA extraction. Five nanograms of total RNA were reverse-transcribed. The resultant cDNA was amplified with 15 cycles using the Taqman PreAmp Master Mix and the Megaplex PreAmp primers, Human Pool A (Life Technologies). miRNAs were measured using the Taqman qPCR Card A v1.0 (Life Technologies).

For mRNA expression, Affymetrix Gene St arrays measured mRNA expression from the same RNA as used for the miRNA measurements. Expression scores were determined by RMA after quantile normalization. These data will be fully described in a separate publication. To determine mRNA targets, Targetscan Release 5.2 [51]and PITA [52] predicted targets were downloaded. Pearson correlations between the RMA scores from Affymetrix Gene St arrays compared to the Taqman qPCR miRNA expression was calculated. Pearson correlations for 1,000 random permutations of equivalently sized gene sets of all non-targeted genes were calculated to determine significance of the predicted mRNA targets. P-values were determined by determining how often the mean of the distribution of the correlation coefficients for each random set was lower or higher than the predicted targets.

### miRNA ISH

miRNAs *in situ* hybridization was performed similar to published protocols [53]. Locked nucleic acids (LNA)-modified probes were 5′ labeled with digoxigenin (Exiqon). After 15 µg/ml proteinase K disgestion, 30 nM of the probe was hybridized to the tissue for 15 hours at 62°C. The probe target was visualized by alkaline phosphatase activity on the nitroblue tetrazolium and bromochloroindoyl phosphate substrate followed by Nuclear Red counterstain. ISH was performed by Exiqon.

### Cell culture and transfections

SKOV-3 was purchased from the American Type Culture Collection. IGROV-1 and OVCAR-8 cell lines were purchased through the National Cancer Institute DTP tumor repository program. Cells were grown in DMEM (Cellgro) with 10% FBS, 1% Penicillin, and 1% streptomycin (Thermo-Fisher) added. Each line was authenticated for genotype and phenotype by the source company. Cells were used at low passage, always less than four months of passaging post-procurement. Cisplatin was purchased from Sigma-Aldrich. Pre-mirs (Life Technologies) and Linked Nucleic Acids (LNAs) (Exiqon) were transfected with Fugene HD (Promega) with indicated concentrations. Negative Control pre-mir mimic 2.0 (Cat #4464058, Life Technologies) and miRCURY LNA(tm) microRNA inhibitor Negative Control A (Cat # 199004, Exiqon).

### Cell Viability, Survival and Spheroid Assays

Cells were plated in 96 well plates and treated with the indicated concentrations of drug 24 hours later. 96 hours after treatment, viability was measured using WST-1 (Roche) according to manufacturer's protocol. Spheroids were grown in micromolds (Microtissues, Providence, RI). The area of the 15 spheroids in the center of each mold was determined in ImageJ. Cells were plated into the molds 24 hours after transfection. Transfected cells were transferred to a 3 cm dish for 24 hours. We then treated them with 30–50 µM Cisplatin, as indicated, for 3 days, with a retreatment 3 days later. After 6–7 days as indicated, viable cells were determined by trypan blue exclusion assay in technical triplicate.

### Affymetrix microarrays

Nugen WT-Ovation Pico kit with the WT-Ovation Exon Module was used to prepare the RNA for Affymetrix Human Gene St v1.0 microarrays following manufacturer instructions in the Brown University Center for Genomics and Proteomics core facility. Data was quantile normalized and signals were estimated using Robust Multi-array Average (RMA). Genes with consistent signal below the lowest quartile were removed. Data are deposited to the Gene Expression Omnibus in series GSE30587.

## Supporting Information

Figure S1 **Tumors are of ovarian origin and are serous epithelial as indicated from examination of H&E and cytokeratin staining.** Representative H&E staining of two representative cases. CA125 and cytokeratin staining of one case is consistent with ovarian tumor origins.
(TIF)

Figure S2 **Bar graph summary of miRNA Taqman expression data shown in** **Figure 1A** **highlighting the miRNA expression changes in each tumor.**
(TIF)

Figure S3 **Taqman assays targeting individual miRNAs and U6 snRNA are consistent with megaplex pooled Taqman assays from bulk tumor purified RNA.** Indiv indicates assay perform with primers only for the designated miRNA. Megaplex is the bulk tumor fold change from the pooled 377 miRNA assay used for the screen shown in Figure 1. All fold changes are calculated using the ΔC_t_ method relative to U6 snRNA. Data for case 1 and 3 are shown.
(TIF)

Figure S4 **miR-146a and miR-150 predicted mRNA targets are significantly down-regulated in omental lesions compared to primary tumors.** **A**. Global distribution of the Pearson correlation coefficients between mRNAs and miRNAs in the primary and metastatic tumors. Red line indicates mRNA targets from the union of TargetScan and PITA predictions. Grey lines are randomly selected sets of transcripts of the same size permuted 1,000 times. P-values are calculated by counting the number of distributions with means lower than the target distribution to define the background. **B**. Genes with Pearson correlation coefficients<−0.3 in the tumors are significantly enriched for specific pathways and functions as determined by Ingenuity Pathway Analysis (IPA). P-values are multiple hypothesis corrected using Benjamini-Hochberg (3). Selected genes for each pathway are listed.
(TIF)

Figure S5 ***In situ*** **hybridization of miR-21 in matched primary tumors and omental metastases.** Cancer cells are stained red by Nuclear Red.
(TIF)

Figure S6 **Pictures of the H&E stained cancer cells before and after laser capture microdissection (LCM).** Pockets of cancer cells were selected for removal and analysis.
(TIF)

Figure S7 **The 8 miRNAs expressed in both cell lines and LCM enriched cancer cells in tumors have increased expression in spheroids compared to monolayer cell culture.** Taqman qPCR array card data of miRNA expression in monolayer and spheroids. Fold changes calculated by ΔCt method normalized to U6 snRNA.
(TIF)

Figure S8 **miR-146a expression after 24 hours after transfection with 50 nM pre-miR in SKOV-3 and OVCAR-8 cells using Taqman assays targeting only miR-146a.** Fold changes calculated by ΔCt method normalized to U6 snRNA. Control are cells transfected with negative control pre-miR. Error bars are standard deviation from three independent experiments transfected in parallel with the functional assays.
(TIF)

Table S1 **Summary of Patient Characteristics.**
(XLSX)

## References

[1] Lengyel E (2010) Ovarian cancer development and metastasis. Am J Pathol 177: 1053–1064. 20651229 10.2353/ajpath.2010.100105 PMC2928939

[2] Paris PL, Hofer MD, Albo G, Kuefer R, Gschwend JE, et al (2006) Genomic profiling of hormone-naive lymph node metastases in patients with prostate cancer. Neoplasia 8: 1083–1089. 17217626 10.1593/neo.06421 PMC1783716

[3] Shah SP, Morin RD, Khattra J, Prentice L, Pugh T, et al (2009) Mutational evolution in a lobular breast tumour profiled at single nucleotide resolution. Nature 461: 809–813. 19812674 10.1038/nature08489

[4] Yachida S, Jones S, Bozic I, Antal T, Leary R, et al (2010) Distant metastasis occurs late during the genetic evolution of pancreatic cancer. Nature 467: 1114–1117. 20981102 10.1038/nature09515 PMC3148940

[5] Bos PD, Zhang XH, Nadal C, Shu W, Gomis RR, et al (2009) Genes that mediate breast cancer metastasis to the brain. Nature 459: 1005–1009. 19421193 10.1038/nature08021 PMC2698953

[6] Ramaswamy S, Ross KN, Lander ES, Golub TR (2003) A molecular signature of metastasis in primary solid tumors. Nat Genet 33: 49–54. 12469122 10.1038/ng1060

[7] Hynes RO (2003) Metastatic potential: generic predisposition of the primary tumor or rare, metastatic variants-or both? Cell 113: 821–823. 12837240 10.1016/s0092-8674(03)00468-9

[8] Nieman KM, Kenny HA, Penicka CV, Ladanyi A, Buell-Gutbrod R, et al (2011) Adipocytes promote ovarian cancer metastasis and provide energy for rapid tumor growth. Nat Med 17: 1498–1503. 22037646 10.1038/nm.2492 PMC4157349

[9] Tredan O, Galmarini CM, Patel K, Tannock IF (2007) Drug resistance and the solid tumor microenvironment. J Natl Cancer Inst 99: 1441–1454. 17895480 10.1093/jnci/djm135

[10] Colella S, Richards KL, Bachinski LL, Baggerly KA, Tsavachidis S, et al (2008) Molecular signatures of metastasis in head and neck cancer. Head Neck 30: 1273–1283. 18642293 10.1002/hed.20871 PMC4136479

[11] Liu CJ, Liu TY, Kuo LT, Cheng HW, Chu TH, et al (2008) Differential gene expression signature between primary and metastatic head and neck squamous cell carcinoma. J Pathol 214: 489–497. 18213732 10.1002/path.2306

[12] Paris PL, Andaya A, Fridlyand J, Jain AN, Weinberg V, et al (2004) Whole genome scanning identifies genotypes associated with recurrence and metastasis in prostate tumors. Hum Mol Genet 13: 1303–1313. 15138198 10.1093/hmg/ddh155

[13] Adib TR, Henderson S, Perrett C, Hewitt D, Bourmpoulia D, et al (2004) Predicting biomarkers for ovarian cancer using gene-expression microarrays. Br J Cancer 90: 686–692. 14760385 10.1038/sj.bjc.6601603 PMC2409606

[14] Hibbs K, Skubitz KM, Pambuccian SE, Casey RC, Burleson KM, et al (2004) Differential gene expression in ovarian carcinoma: identification of potential biomarkers. Am J Pathol 165: 397–414. 15277215 10.1016/S0002-9440(10)63306-8 PMC1618570

[15] Lancaster JM, Dressman HK, Clarke JP, Sayer RA, Martino MA, et al (2006) Identification of genes associated with ovarian cancer metastasis using microarray expression analysis. Int J Gynecol Cancer 16: 1733–1745. 17009964 10.1111/j.1525-1438.2006.00660.x

[16] Hudson LG, Zeineldin R, Stack MS (2008) Phenotypic plasticity of neoplastic ovarian epithelium: unique cadherin profiles in tumor progression. Clin Exp Metastasis 25: 643–655. 18398687 10.1007/s10585-008-9171-5 PMC2836537

[17] Cowden Dahl KD, Symowicz J, Ning Y, Gutierrez E, Fishman DA, et al (2008) Matrix metalloproteinase 9 is a mediator of epidermal growth factor-dependent e-cadherin loss in ovarian carcinoma cells. Cancer Res 68: 4606–4613. 18559505 10.1158/0008-5472.CAN-07-5046 PMC3621086

[18] Moss NM, Barbolina MV, Liu Y, Sun L, Munshi HG, et al (2009) Ovarian cancer cell detachment and multicellular aggregate formation are regulated by membrane type 1 matrix metalloproteinase: a potential role in I.p. metastatic dissemination. Cancer Res 69: 7121–7129. 19706774 10.1158/0008-5472.CAN-08-4151 PMC2737080

[19] Sawada K, Mitra AK, Radjabi AR, Bhaskar V, Kistner EO, et al (2008) Loss of E-cadherin promotes ovarian cancer metastasis via alpha 5-integrin, which is a therapeutic target. Cancer Res 68: 2329–2339. 18381440 10.1158/0008-5472.CAN-07-5167 PMC2665934

[20] Chen C, Ridzon DA, Broomer AJ, Zhou Z, Lee DH, et al (2005) Real-time quantification of microRNAs by stem-loop RT-PCR. Nucleic Acids Res 33: e179. 16314309 10.1093/nar/gni178 PMC1292995

[21] Creighton CJ, Hernandez-Herrera A, Jacobsen A, Levine DA, Mankoo P, et al (2012) Integrated analyses of microRNAs demonstrate their widespread influence on gene expression in high-grade serous ovarian carcinoma. PLoS One 7: e34546. 22479643 10.1371/journal.pone.0034546 PMC3315571

[22] Grun B, Benjamin E, Sinclair J, Timms JF, Jacobs IJ, et al (2009) Three-dimensional in vitro cell biology models of ovarian and endometrial cancer. Cell Prolif 42: 219–228. 19222485 10.1111/j.1365-2184.2008.00579.x PMC6496843

[23] Weigelt B, Lo AT, Park CC, Gray JW, Bissell MJ (2010) HER2 signaling pathway activation and response of breast cancer cells to HER2-targeting agents is dependent strongly on the 3D microenvironment. Breast Cancer Res Treat 122: 35–43. 19701706 10.1007/s10549-009-0502-2 PMC2935800

[24] Rizvi I, Celli JP, Evans CL, Abu-Yousif AO, Muzikansky A, et al (2010) Synergistic enhancement of carboplatin efficacy with photodynamic therapy in a three-dimensional model for micrometastatic ovarian cancer. Cancer Res 70: 9319–9328. 21062986 10.1158/0008-5472.CAN-10-1783 PMC3057933

[25] Rahmanzadeh R, Rai P, Celli JP, Rizvi I, Baron-Luhr B, et al (2010) Ki-67 as a molecular target for therapy in an in vitro three-dimensional model for ovarian cancer. Cancer Res 70: 9234–9242. 21045152 10.1158/0008-5472.CAN-10-1190 PMC3057762

[26] Muranen T, Selfors LM, Worster DT, Iwanicki MP, Song L, et al (2012) Inhibition of PI3K/mTOR Leads to Adaptive Resistance in Matrix-Attached Cancer Cells. Cancer Cell 21: 227–239. 22340595 10.1016/j.ccr.2011.12.024 PMC3297962

[27] Shield K, Ackland ML, Ahmed N, Rice GE (2009) Multicellular spheroids in ovarian cancer metastases: Biology and pathology. Gynecol Oncol 113: 143–148. 19135710 10.1016/j.ygyno.2008.11.032

[28] Napolitano AP, Dean DM, Man AJ, Youssef J, Ho DN, et al (2007) Scaffold-free three-dimensional cell culture utilizing micromolded nonadhesive hydrogels. Biotechniques 43: 494, 496–500. 18019341 10.2144/000112591

[29] Sharma SV, Lee DY, Li B, Quinlan MP, Takahashi F, et al (2010) A chromatin-mediated reversible drug-tolerant state in cancer cell subpopulations. Cell 141: 69–80. 20371346 10.1016/j.cell.2010.02.027 PMC2851638

[30] Krichevsky AM, Gabriely G (2009) miR-21: a small multi-faceted RNA. J Cell Mol Med 13: 39–53. 19175699 10.1111/j.1582-4934.2008.00556.x PMC3823035

[31] Baek D, Villen J, Shin C, Camargo FD, Gygi SP, et al (2008) The impact of microRNAs on protein output. Nature 455: 64–71. 18668037 10.1038/nature07242 PMC2745094

[32] Mukherji S, Ebert MS, Zheng GX, Tsang JS, Sharp PA, et al (2011) MicroRNAs can generate thresholds in target gene expression. Nat Genet 43: 854–859. 21857679 10.1038/ng.905 PMC3163764

[33] Xiao C, Calado DP, Galler G, Thai TH, Patterson HC, et al (2007) MiR-150 controls B cell differentiation by targeting the transcription factor c-Myb. Cell 131: 146–159. 17923094 10.1016/j.cell.2007.07.021

[34] Zhou B, Wang S, Mayr C, Bartel DP, Lodish HF (2007) miR-150, a microRNA expressed in mature B and T cells, blocks early B cell development when expressed prematurely. Proc Natl Acad Sci U S A 104: 7080–7085. 17438277 10.1073/pnas.0702409104 PMC1855395

[35] Wu Q, Jin H, Yang Z, Luo G, Lu Y, et al (2010) MiR-150 promotes gastric cancer proliferation by negatively regulating the pro-apoptotic gene EGR2. Biochem Biophys Res Commun 392: 340–345. 20067763 10.1016/j.bbrc.2009.12.182

[36] Li YJ, Zhang YX, Wang PY, Chi YL, Zhang C, et al (2011) Regression of A549 lung cancer tumors by anti-miR-150 vector. Oncol Rep 10.3892/or.2011.1466 21935578

[37] Ma Y, Zhang P, Wang F, Zhang H, Yang J, et al (2011) miR-150 as a potential biomarker associated with prognosis and therapeutic outcome in colorectal cancer. Gut 10.1136/gutjnl-2011-301122 22052060

[38] Watanabe A, Tagawa H, Yamashita J, Teshima K, Nara M, et al (2011) The role of microRNA-150 as a tumor suppressor in malignant lymphoma. Leukemia 25: 1324–1334. 21502955 10.1038/leu.2011.81

[39] Lovat F, Valeri N, Croce CM (2011) MicroRNAs in the pathogenesis of cancer. Semin Oncol 38: 724–733. 22082758 10.1053/j.seminoncol.2011.08.006

[40] Garzon R, Marcucci G, Croce CM (2010) Targeting microRNAs in cancer: rationale, strategies and challenges. Nat Rev Drug Discov 9: 775–789. 20885409 10.1038/nrd3179 PMC3904431

[41] Zhou L, Qi X, Potashkin JA, Abdul-Karim FW, Gorodeski GI (2008) MicroRNAs miR-186 and miR-150 down-regulate expression of the pro-apoptotic purinergic P2X7 receptor by activation of instability sites at the 3'-untranslated region of the gene that decrease steady-state levels of the transcript. J Biol Chem 283: 28274–28286. 18682393 10.1074/jbc.M802663200 PMC2568908

[42] Taganov KD, Boldin MP, Chang KJ, Baltimore D (2006) NF-kappaB-dependent induction of microRNA miR-146, an inhibitor targeted to signaling proteins of innate immune responses. Proc Natl Acad Sci U S A 103: 12481–12486. 16885212 10.1073/pnas.0605298103 PMC1567904

[43] Bhaumik D, Scott GK, Schokrpur S, Patil CK, Campisi J, et al (2008) Expression of microRNA-146 suppresses NF-kappaB activity with reduction of metastatic potential in breast cancer cells. Oncogene 27: 5643–5647. 18504431 10.1038/onc.2008.171 PMC2811234

[44] Garcia AI, Buisson M, Bertrand P, Rimokh R, Rouleau E, et al (2011) Down-regulation of BRCA1 expression by miR-146a and miR-146b-5p in triple negative sporadic breast cancers. EMBO Mol Med 3: 279–290. 21472990 10.1002/emmm.201100136 PMC3377076

[45] Suzuki Y, Kim HW, Ashraf M, Haider H (2010) Diazoxide potentiates mesenchymal stem cell survival via NF-kappaB-dependent miR-146a expression by targeting Fas. Am J Physiol Heart Circ Physiol 299: H1077–1082. 20656888 10.1152/ajpheart.00212.2010 PMC2957349

[46] Borst P, Rottenberg S, Jonkers J (2008) How do real tumors become resistant to cisplatin? Cell Cycle 7: 1353–1359. 18418074 10.4161/cc.7.10.5930

[47] Casey RC, Burleson KM, Skubitz KM, Pambuccian SE, Oegema TR Jr, et al (2001) Beta 1-integrins regulate the formation and adhesion of ovarian carcinoma multicellular spheroids. Am J Pathol 159: 2071–2080. 11733357 10.1016/s0002-9440(10)63058-1 PMC1850600

[48] Zietarska M, Maugard CM, Filali-Mouhim A, Alam-Fahmy M, Tonin PN, et al (2007) Molecular description of a 3D in vitro model for the study of epithelial ovarian cancer (EOC). Mol Carcinog 46: 872–885. 17455221 10.1002/mc.20315

[49] Sodek KL, Ringuette MJ, Brown TJ (2009) Compact spheroid formation by ovarian cancer cells is associated with contractile behavior and an invasive phenotype. Int J Cancer 124: 2060–2070. 19132753 10.1002/ijc.24188

[50] Gene-E website. Available: http://www.broadinstitute.org/cancer/software/GENE-E/. Accessed 2013 Feb 18.

[51] Lewis BP, Burge CB, Bartel DP (2005) Conserved seed pairing, often flanked by adenosines, indicates that thousands of human genes are microRNA targets. Cell 120: 15–20. 15652477 10.1016/j.cell.2004.12.035

[52] Kertesz M, Ivino N, Unnerstall U, Gaul U, Segal E (2007) The role of site accessibility in microRNA target recognition. Nat Genet 39: 1278–1284. 17893677 10.1038/ng2135

[53] Nielsen BS (2012) MicroRNA in situ hybridization. Methods Mol Biol 822: 67–84. 22144192 10.1007/978-1-61779-427-8_5

