# Correction: Identification of Ovarian Cancer Metastatic miRNAs

**DOI:** 10.1371/annotation/aeb6ccee-921a-4250-a18c-4ea633b984dd

**Published:** 2014-01-03

**Authors:** Souriya Vang, Hsin-Ta Wu, Andrew Fischer, Daniel H. Miller, Shannon MacLaughlan, Elijah Douglass, Margaret Steinhoff, Colin Collins, Peter J. S. Smith, Laurent Brard, Alexander S. Brodsky

Lauren Comisar was erroneously omitted from the author list. Her affiliation is: Department of Molecular Biology, Cell Biology, and Biochemistry, Brown University.

The updated author list is:

Souriya Vang, Hsin-Ta Wu, Andrew Fischer, Daniel H. Miller, Shannon MacLaughlan, Elijah Douglass, Lauren Comisar, Margaret Steinhoff, Colin Collins,Peter J. S. Smith, Laurent Brard, Alexander S. Brodsky

The updated author contributions are:

Pathology and tissue analysis: MS. Funding and intellectual support: CC PJS. Conceived and designed the experiments: ASB LB. Performed the experiments: SV AF DHM ED LC. Analyzed the data: HTW ASB. Contributed reagents/materials/analysis tools: SM. Wrote the paper: ASB. 

