# Supplementary figures and images for: Identification of Ovarian Cancer Metastatic miRNAs
Source: PLoS One. 2013 Mar 12;8(3):e58226. doi: 10.1371/journal.pone.0058226 (PMC3595263; doi:10.1371/journal.pone.0058226)

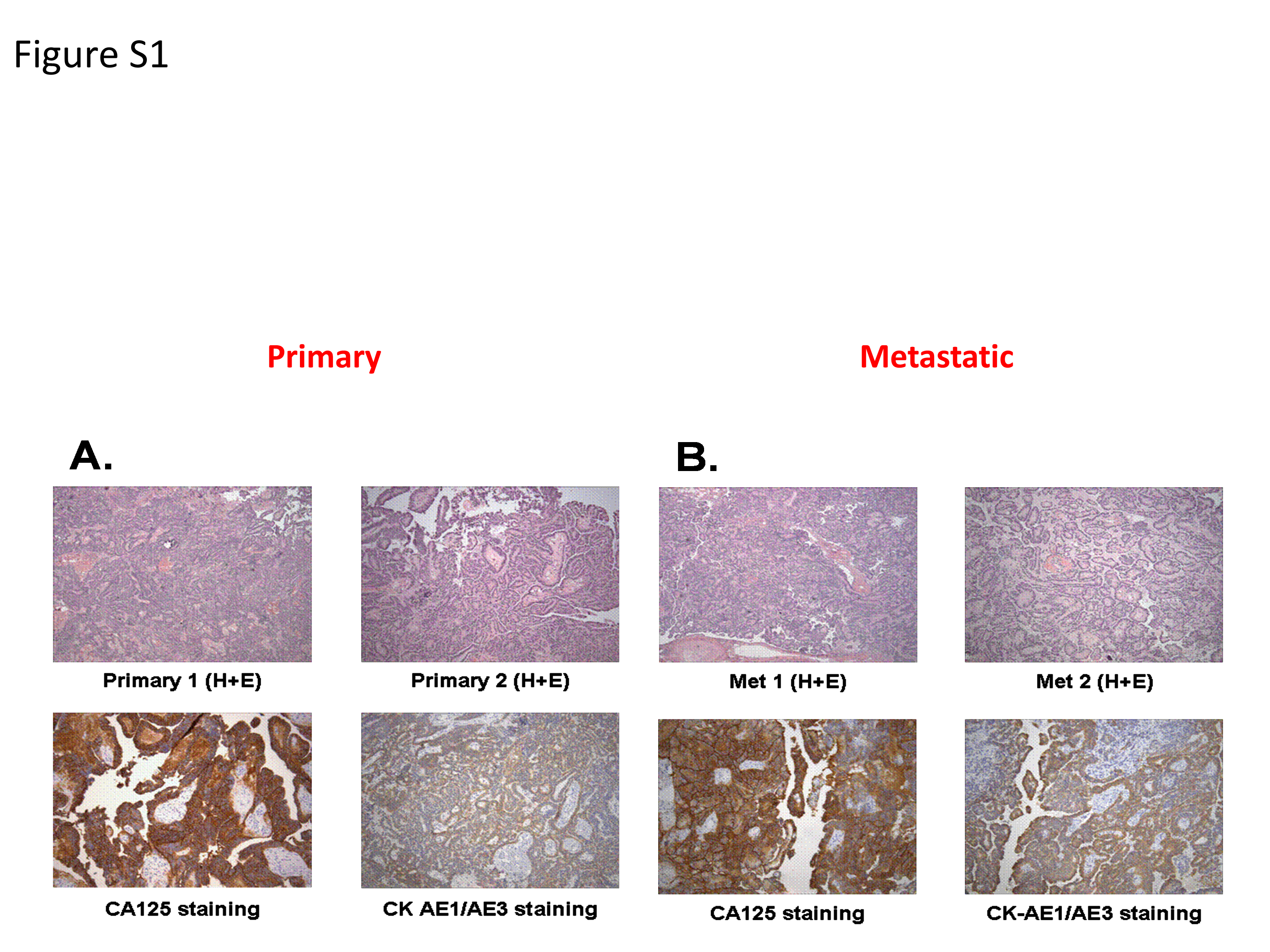

Supplement: Figure S1 — Tumors are of ovarian origin and are serous epithelial as indicated from examination of H&E and cytokeratin staining. Representative H&E staining of two representative cases. CA125 and cytokeratin staining of one case is consistent with ovarian tumor origins. (TIF) [file pone.0058226.s001.tif]

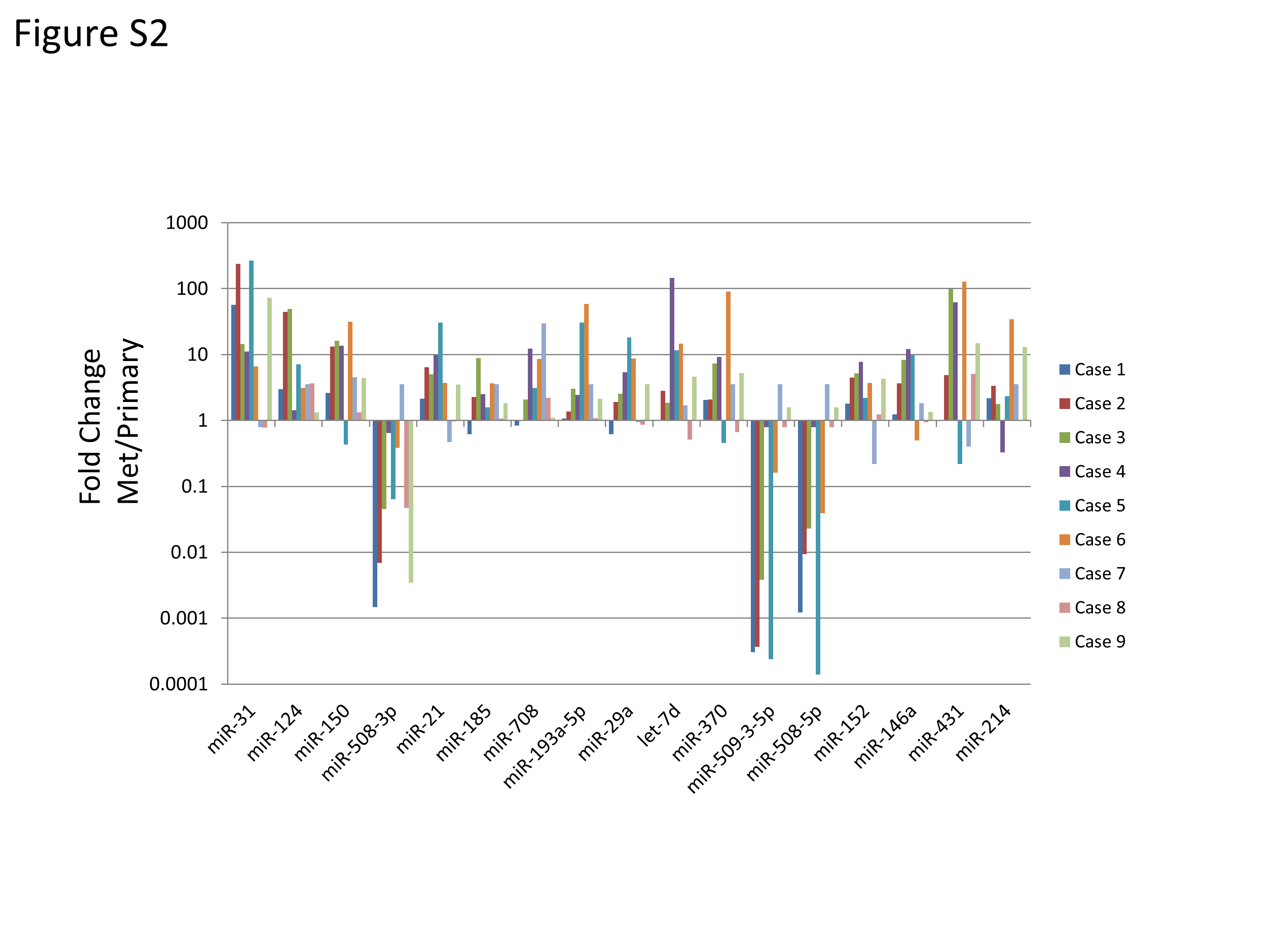

Supplement: Figure S2 — Bar graph summary of miRNA Taqman expression data shown in Figure 1A highlighting the miRNA expression changes in each tumor. (TIF) [file pone.0058226.s002.tif]

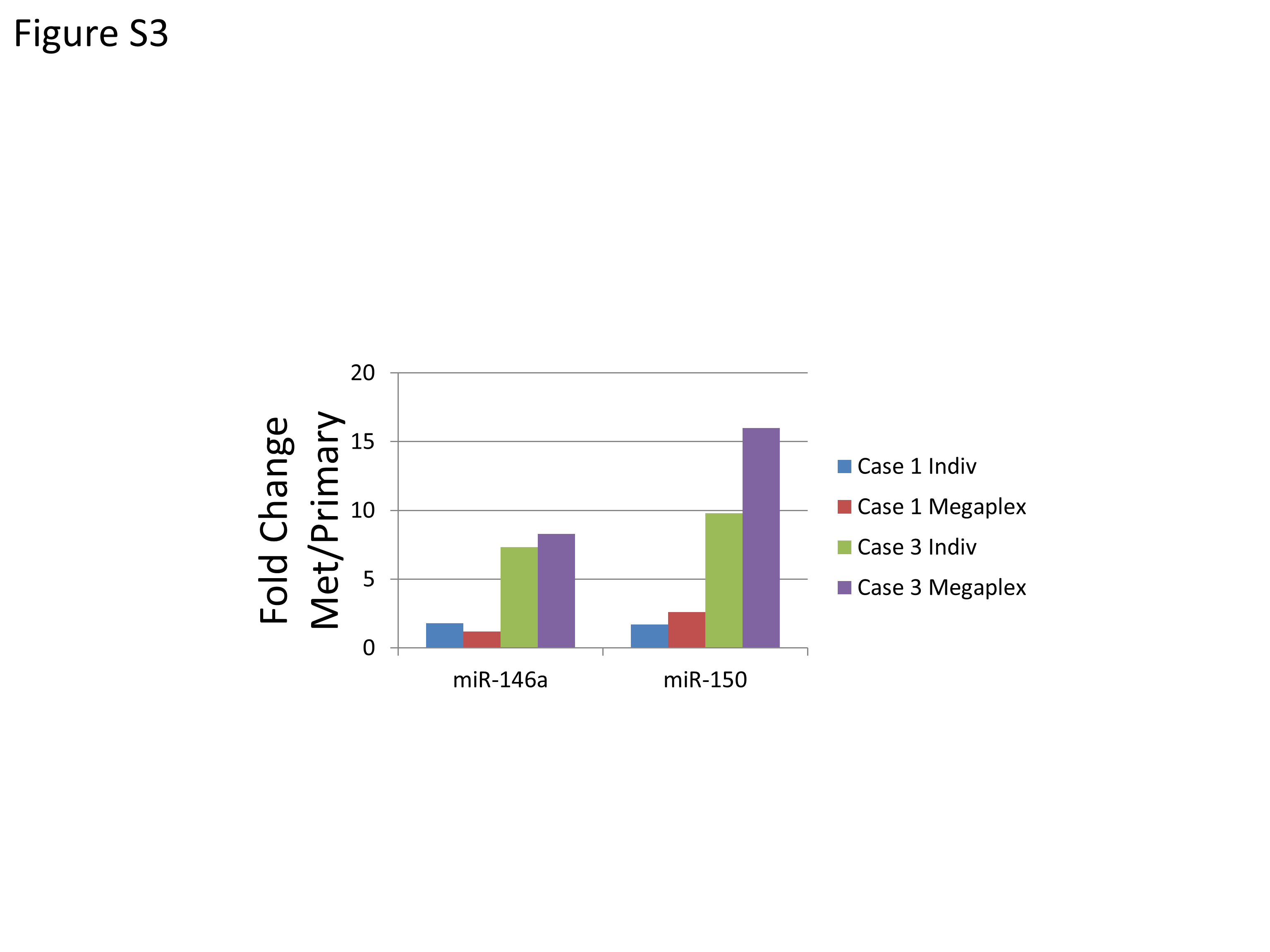

Supplement: Figure S3 — Taqman assays targeting individual miRNAs and U6 snRNA are consistent with megaplex pooled Taqman assays from bulk tumor purified RNA. Indiv indicates assay perform with primers only for the designated miRNA. Megaplex is the bulk tumor fold change from the pooled 377 miRNA assay used for the screen shown in Figure 1. All fold changes are calculated using the ΔCt method relative to U6 snRNA. Data for case 1 and 3 are shown. (TIF) [file pone.0058226.s003.tif]

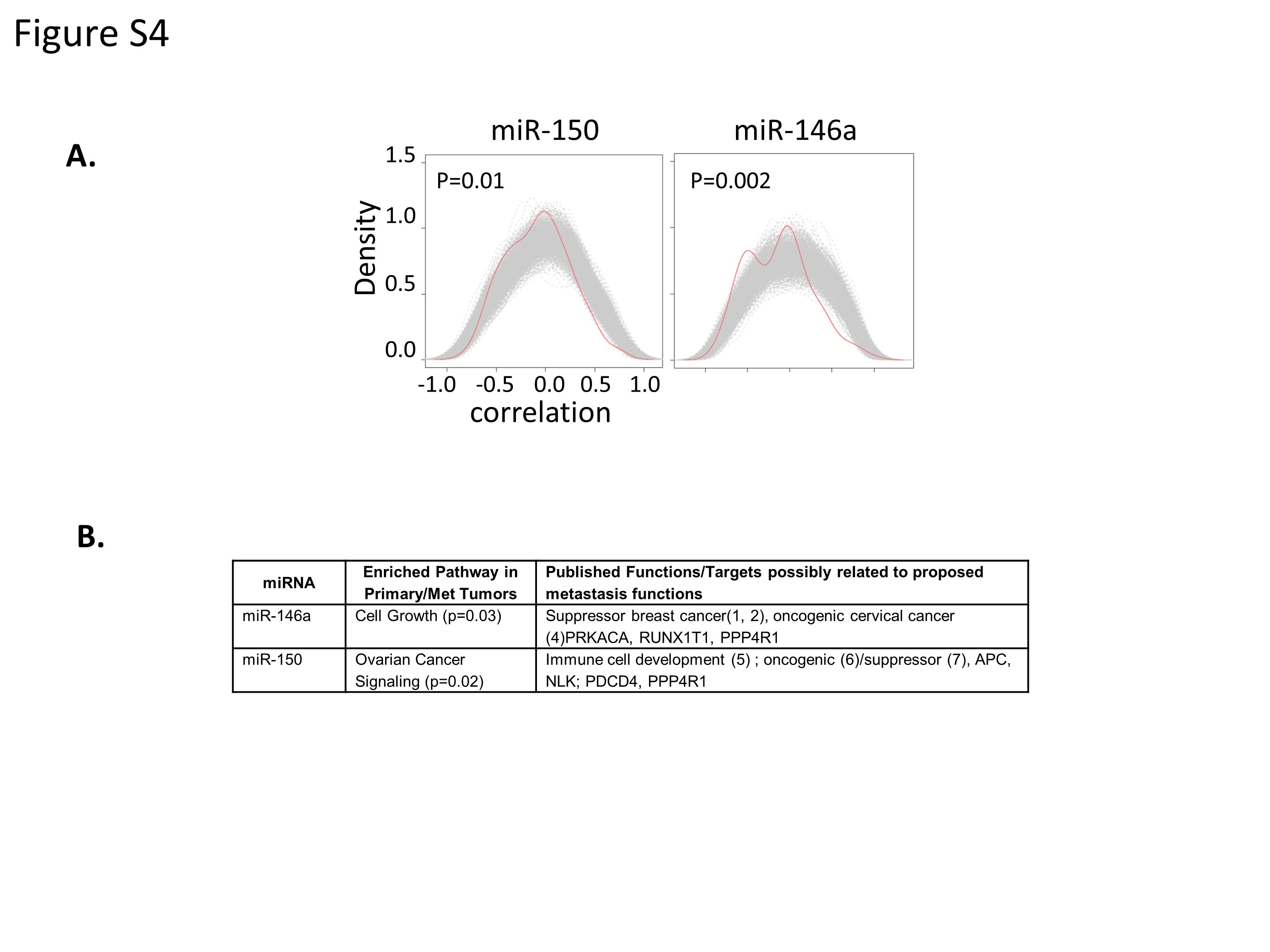

Supplement: Figure S4 — miR-146a and miR-150 predicted mRNA targets are significantly down-regulated in omental lesions compared to primary tumors. A. Global distribution of the Pearson correlation coefficients between mRNAs and miRNAs in the primary and metastatic tumors. Red line indicates mRNA targets from the union of TargetScan and PITA predictions. Grey lines are randomly selected sets of transcripts of the same size permuted 1,000 times. P-values are calculated by counting the number of distributions with means lower than the target distribution to define the background. B. Genes with Pearson correlation coefficients<−0.3 in the tumors are significantly enriched for specific pathways and functions as determined by Ingenuity Pathway Analysis (IPA). P-values are multiple hypothesis corrected using Benjamini-Hochberg (3). Selected genes for each pathway are listed. (TIF) [file pone.0058226.s004.tif]

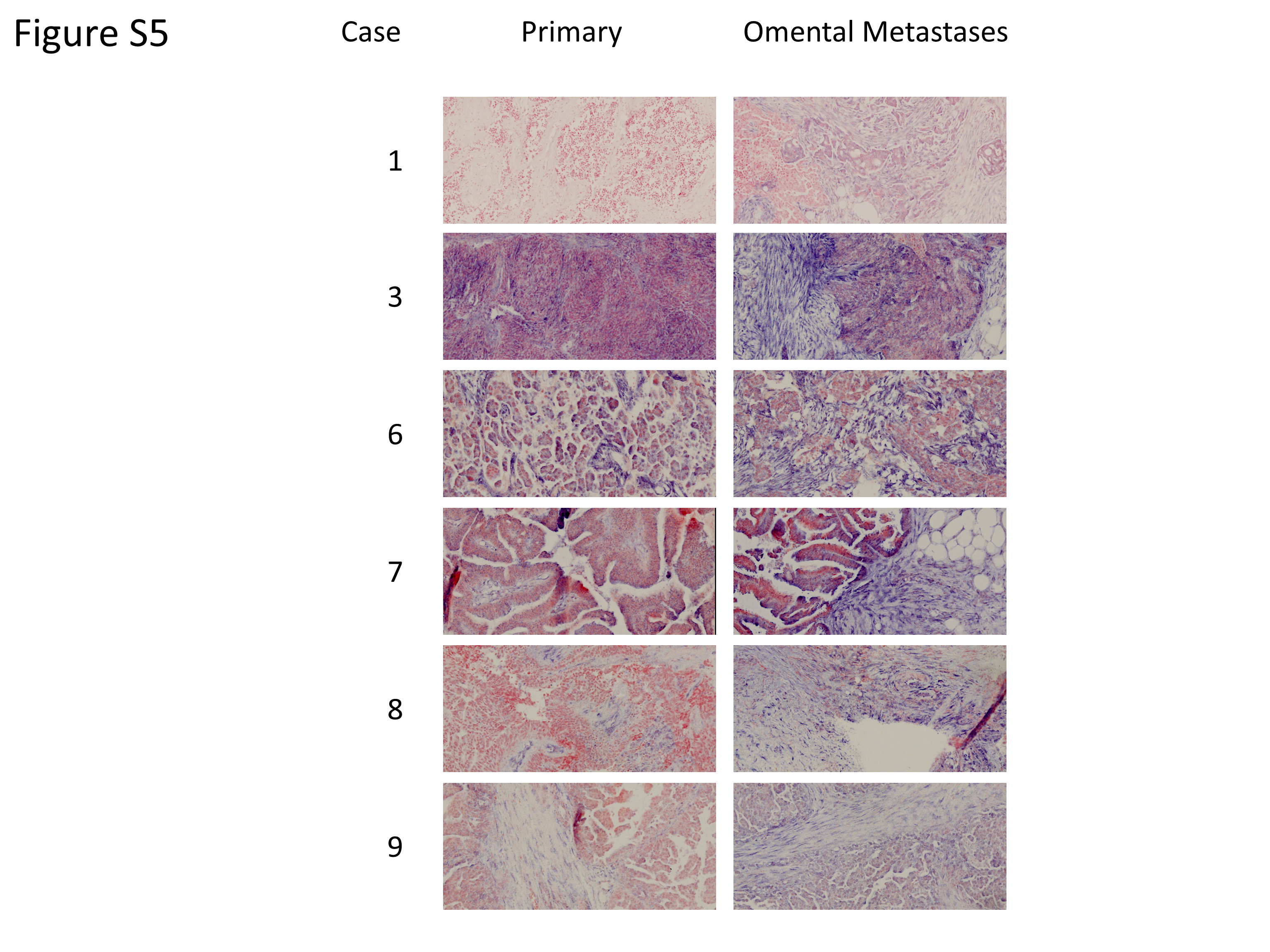

Supplement: Figure S5 — In situ hybridization of miR-21 in matched primary tumors and omental metastases. Cancer cells are stained red by Nuclear Red. (TIF) [file pone.0058226.s005.tif]

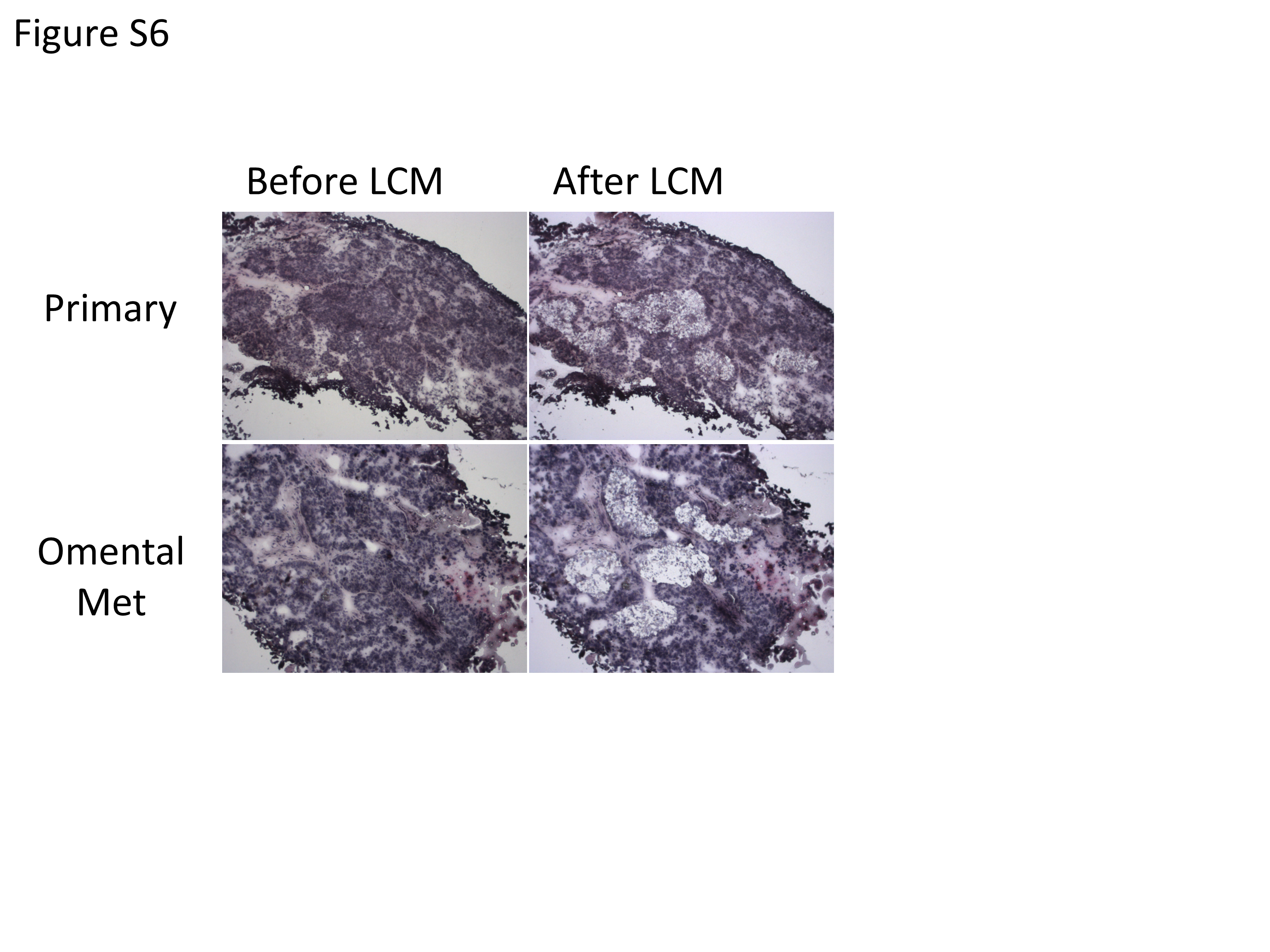

Supplement: Figure S6 — Pictures of the H&E stained cancer cells before and after laser capture microdissection (LCM). Pockets of cancer cells were selected for removal and analysis. (TIF) [file pone.0058226.s006.tif]

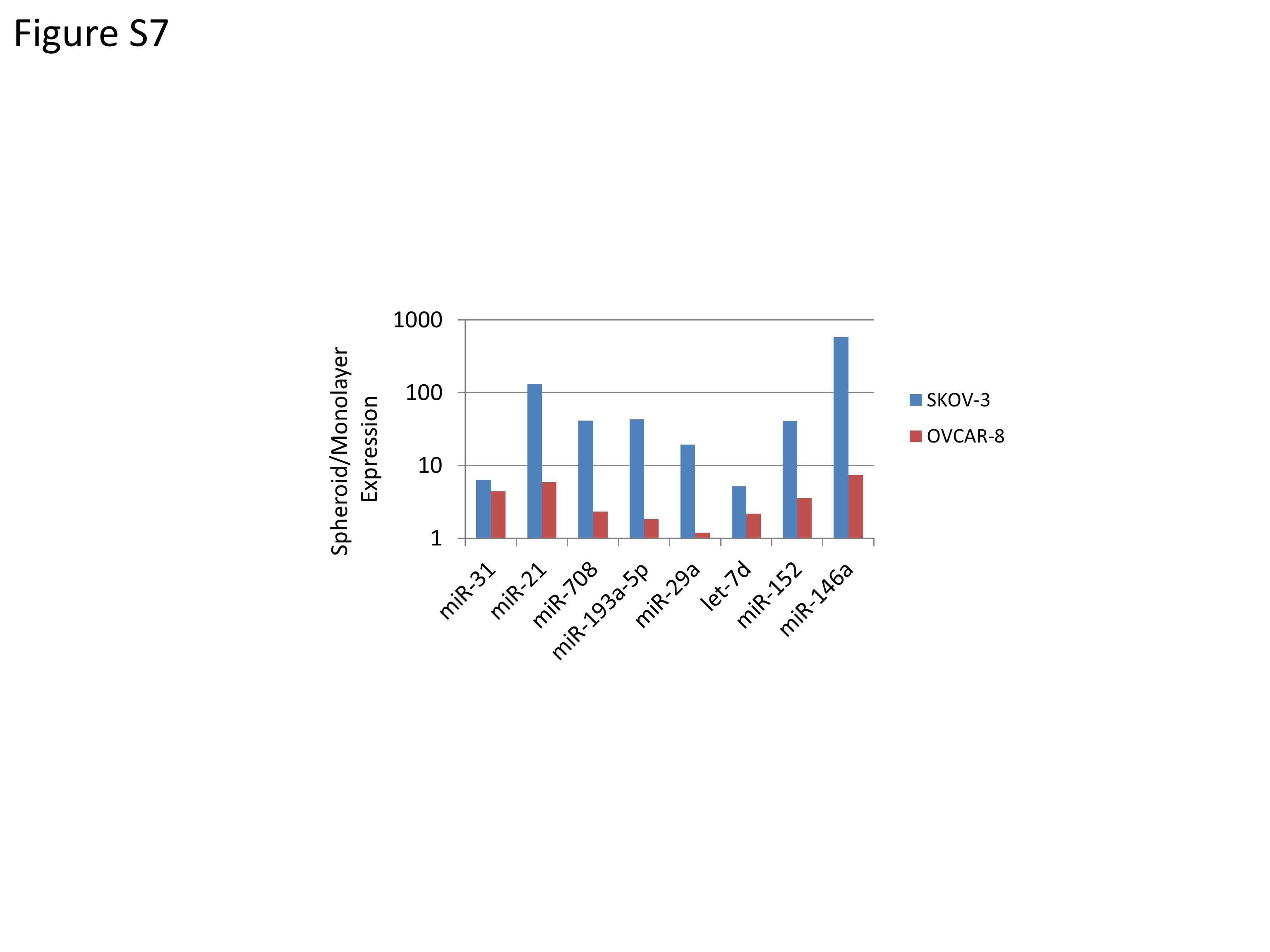

Supplement: Figure S7 — The 8 miRNAs expressed in both cell lines and LCM enriched cancer cells in tumors have increased expression in spheroids compared to monolayer cell culture. Taqman qPCR array card data of miRNA expression in monolayer and spheroids. Fold changes calculated by ΔCt method normalized to U6 snRNA. (TIF) [file pone.0058226.s007.tif]

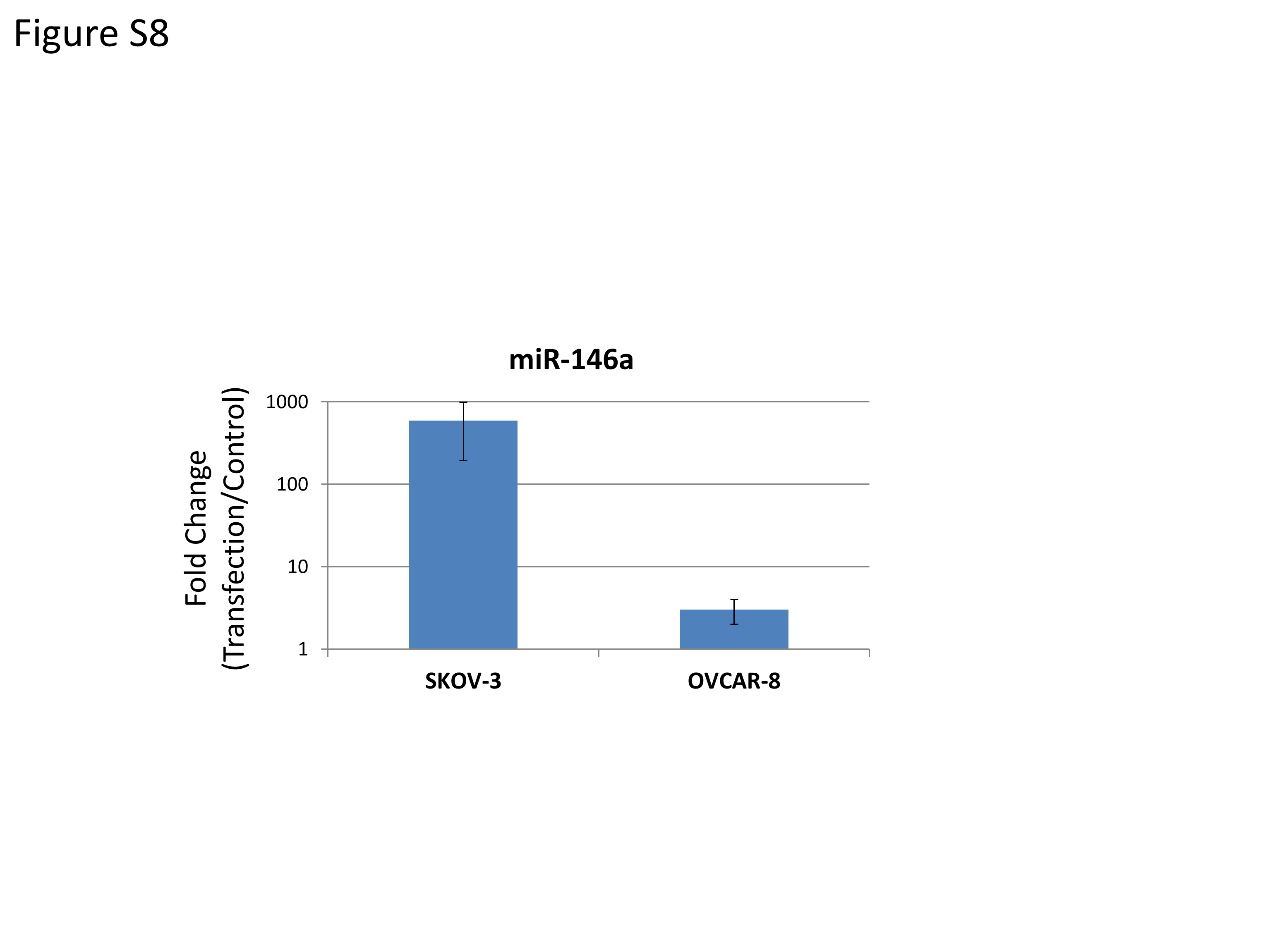

Supplement: Figure S8 — miR-146a expression after 24 hours after transfection with 50 nM pre-miR in SKOV-3 and OVCAR-8 cells using Taqman assays targeting only miR-146a. Fold changes calculated by ΔCt method normalized to U6 snRNA. Control are cells transfected with negative control pre-miR. Error bars are standard deviation from three independent experiments transfected in parallel with the functional assays. (TIF) [file pone.0058226.s008.tif]
